# The Drinking Habit

**Published:** 1890-03

**Authors:** 


### THE DRINKING HABIT.

Statistics show that among intemperate persons between the ages of twenty and thirty the mortality is five times greater than among temperate persons. From thirty to fifty the mortality is four times greater with the intemperate, and from fifty to sixty it is three times greater, while from sixty to eighty it is twice as great. These are figures that do not lie, and old topers and moderate drinkers should take a hint. In a group of total abstainers, aged twenty, the average of life left is forty-four and two-tenths years, while with moderate drinkers the average would be fifteen and six-tenths years. That is to say, a total abstainer on an average would live to be sixty-four, while the moderate drinker would be cut off at thirty-five. A drinker is more liable to accidental death than a sober man is, and in addition to that he is steadily breaking down his constitution.

